# Transcriptional Priming of *Salmonella* Pathogenicity Island-2 Precedes Cellular Invasion

**DOI:** 10.1371/journal.pone.0021648

**Published:** 2011-06-28

**Authors:** Suzanne E. Osborne, Brian K. Coombes

**Affiliations:** Department of Biochemistry and Biomedical Sciences, Michael G. DeGroote Institute for Infectious Disease Research, McMaster University, Hamilton, Ontario, Canada; Institut de Pharmacologie et de Biologie Structurale, France

## Abstract

Invasive salmonellosis caused by *Salmonella enterica* involves an enteric stage of infection where the bacteria colonize mucosal epithelial cells, followed by systemic infection with intracellular replication in immune cells. The type III secretion system encoded in *Salmonella* Pathogenicity Island (SPI)-2 is essential for intracellular replication and the regulators governing high-level expression of SPI-2 genes within the macrophage phagosome and in inducing media thought to mimic this environment have been well characterized. However, low-level expression of SPI-2 genes is detectable in media thought to mimic the extracellular environment suggesting that additional regulatory pathways are involved in SPI-2 gene expression prior to cellular invasion. The regulators involved in this activity are not known and the extracellular transcriptional activity of the entire SPI-2 island *in vivo* has not been studied. We show that low-level, SsrB-independent promoter activity for the *ssrA-ssrB* two-component regulatory system and the *ssaG* structural operon encoded in SPI-2 is dependent on transcriptional input by OmpR and Fis under non-inducing conditions. Monitoring the activity of all SPI-2 promoters in real-time following oral infection of mice revealed invasion-independent transcriptional activity of the SPI2 T3SS in the lumen of the gut, which we suggest is a priming activity with functional relevance for the subsequent intracellular host-pathogen interaction.

## Introduction

*Salmonella enterica* causes a range of foodborne diseases from self-limiting gastroenteritis to fatal systemic infections. The virulence capabilities of *Salmonella* is mediated by two type III secretion systems (T3SS) which function to deliver bacterial proteins, called effectors, into host cells that can reprogram various aspects of host biology [1], [2]. The two T3SS in *Salmonella* are encoded by separate horizontally acquired pathogenicity islands termed *Salmonella* Pathogenicity Island (SPI)-1 and SPI-2. The T3SS-1 allows *Salmonella* to invade into host epithelial cells and is needed to establish infection in the gastrointestinal tract [3]. Following passage across the host epithelial barrier the bacteria are engulfed by resident immune cells, chiefly macrophages [4], [5], and induce the expression of the T3SS-2 [6], [7]. Effectors translocated by the T3SS-2 play a critical role in protection against an arsenal of host defences including recruitment of reactive oxygen (ROS) and reactive nitrogen (RNS) species to the *Salmonella* containing vacuole (SCV) [8], [9].

The T3SS in SPI-2 is organized into four major operons; a regulatory operon, a structural-1 operon, an effector/chaperone operon and a structural-2 operon. Genes in these operons are controlled by promoters in front of *ssrA*, *ssaB*, *sseA* and *ssaG* respectively [10], [11]. We recently identified two additional promoters (*ssaM* and *ssaR*) in the structural-2 operon [11]. The major regulator of SPI-2 gene expression is a two-component regulatory system encoded by the genes *ssrA* and *ssrB* in the linked regulatory operon. In response to an unidentified environmental cue, the SsrA sensor kinase autophosphoryates and activates the SsrB response regulator that can bind to an evolved palindrome sequence to induce gene expression from the SPI-2 promoters and at several promoters outside of SPI-2 [11], [12]. Expression of *ssrA* and *ssrB* is autoregulated and also dependent on several transcription factors including the two-component systems PhoP-PhoQ, OmpR-EnvZ, as well as SlyA and Fis. SPI-2 is negatively regulated by H-NS, Hha and YdgT [13], [14], [15], [16], [17], [18]. 

It is well established that transcriptional activity in SPI-2 is induced following intracellular invasion as well as in *in vitro* conditions thought to mimic the intracellular environment [19], [20], [21]. However, we and others have reported low-level SPI-2 gene expression in non-inducing media that does not simulate the intracellular environment [21], [22], [23]. Of particular importance is that the expression under non-inducing conditions is independent of SsrB, suggesting another transcriptional input pathway for SPI-2 gene expression that may precede cellular invasion. In addition to its role in systemic dissemination of bacteria, accumulating evidence indicates that the SPI-2 T3SS facilitates bacterial colonization of the gut and induces intestinal inflammation [24], [25], [26]. It was also shown using recombinase-based *in vivo* expression that three promoters in SPI-2 (*sseA, ssaG* and *spiC/ssaB*) are activated within 15 min after entering mouse ileal loops [27]. These data suggest that a transcriptional regulatory circuit operates to induce low-level gene expression in SPI-2 prior to *Salmonella*'s invasion into host cells.

We analyzed the activity of all six promoters in SPI-2 in both inducing and non-inducing media in a variety of *Salmonella* mutants lacking the regulators involved in SPI-2 gene expression. Inducing media resulted in high simultaneous activity of each SPI-2 promoter that was dependent on SsrB. In contrast, SPI-2 promoters had low-level activity in non-inducing media that was independent of SsrB but instead dependent on OmpR or Fis. We further analyzed SPI-2 promoter activity during animal infection in real time and found that SPI-2 promoters were activated immediately following entry into the small intestine that was independent of invasion. Using cultured epithelial cells we demonstrate that SPI-2 has two distinct activation steps; an initial activation that precedes cellular invasion, followed by the classical intracellular activation pathway for high-level induction.

## Results

### Regulation of SPI-2 under non-inducing conditions

**Figure 1:**
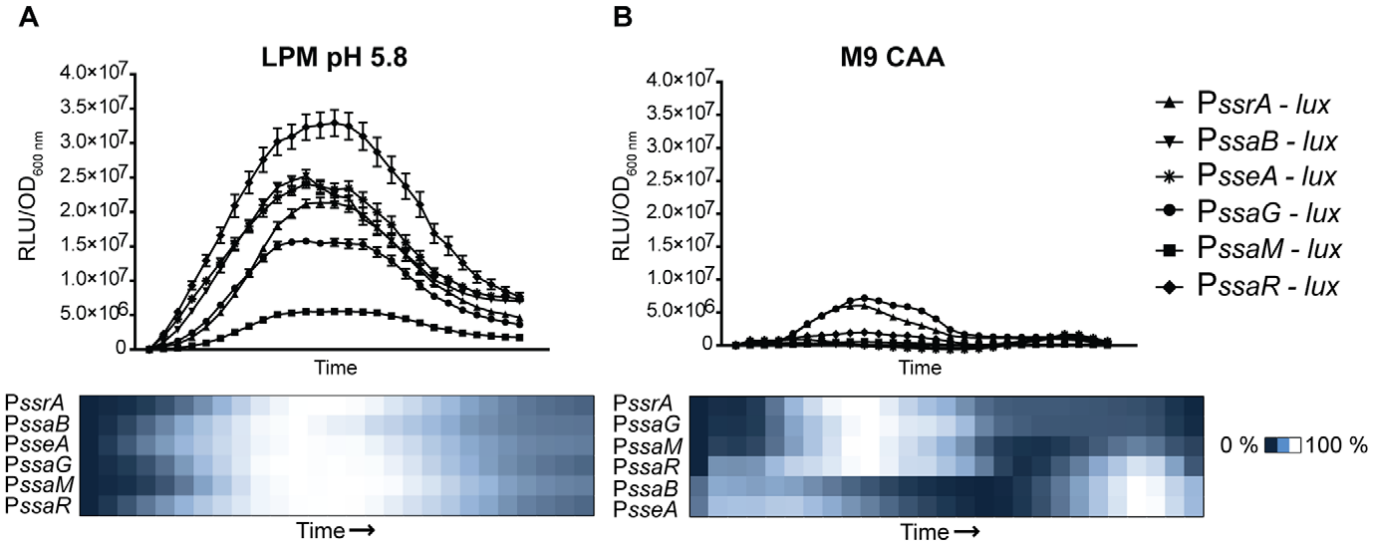
SPI-2 expression in inducing and non-inducing conditions *in vitro*. *S*. Typhimurium with luciferase transcriptional reporters for each SPI-2 promoter were sub-cultured from actively growing cultures in M9-CAA into either (**A**) inducing (LPM pH 5.8) or (**B**) non-inducing (M9-CAA) media. Luminescence was quantified continuously and normalized to OD_600 nm_ at each time point (*n* = 12). Heat maps represent the percent activity relative to each individual promoter's maximal expression level.

To compare the activity of SPI-2 promoters in both inducing and non-inducing conditions *in vitro* we constructed bacterial luciferase transcriptional reporters for each of the six promoters in SPI-2 (*ssrA*, *ssaB*, *sseA*, *ssaG*, *ssaM* and *ssaR*) [10], [11]. To simulate inducing conditions we used an acidic minimal medium low in phosphate and magnesium (LPM pH 5.8) that is well established to activate robust SPI-2 gene expression [21]. M9-CAA medium containing millimolar concentrations of divalent cations and a neutral pH was used as a non-inducing media [20]. Wild type *S*. Typhimurium containing transcriptional reporters were grown in M9-CAA until mid-log phase at which point they were sub-cultured into either inducing or non-inducing media followed by continuous luminescence measurements. Following transfer to LPM each SPI-2 promoter was induced with the same kinetics but the magnitude of this activity varied with each promoter (Figure 1A). Promoter activity peaked at early to mid-exponential phase and then declined and remained constant at ∼20–30% of maximum activity (Table S1 for complete dataset). We consistently observed an early, low-level promoter activity primarily from the regulatory and structural-1 promoters (*ssrA* and *ssaG*) under non-inducing conditions followed by delayed activity from the remaining structural-2 and effector promoters (Figure 1B). These results suggested that SPI-2 promoters had unique transcriptional inputs under inducing and non-inducing conditions that gave rise to differential timing and magnitude of gene expression.

### SPI-2 expression in non-inducing conditions has distinct regulatory inputs

In order to understand the regulatory input contributing to the activity of SPI-2 promoters in non-inducing conditions, we measured promoter activity in eight different mutants each lacking a major regulator known to be involved in virulence gene expression in *Salmonella* including *ssrB*, *ompR*, *slyA*, *phoP*, *fis*, *ydgT*, *hha* and *hns* [11], [17], [18], [28], [29], [30]. Loss of SsrB, OmpR, SlyA, PhoP or Fis caused a marked decrease in the promoter activity observed in LPM for each SPI-2 promoter (Table 1; Figures S1, S2, Table S1 for full dataset). Interestingly, loss of PhoP altered the temporal dynamics of all promoters with the exception of the *ssrA* promoter (Figure S1). Deletion of the SPI-2 repressors YdgT, Hha or expression of dominant-negative H-NS (HNSQ92am) [31] increased SPI-2 promoter activity in most cases although loss of YdgT and Hha caused a decrease in *ssaG* and *ssaR* promoter activity (Figure S2). The *ssrA* and *ssaG* promoter activity in M9-CAA was independent of SsrB. Instead, the *ssrA* promoter activity in M9-CAA was dependent on OmpR and partially dependent on Fis, whereas *ssaG* promoter activity was dependent only on Fis. These results confirmed that the low-level SPI-2 promoter activity under non-inducing conditions had regulatory inputs distinct from that needed for high-level expression under inducing conditions thought to mimic the intracellular environment.

**Table 1:** Transcriptional reporter activity in various mutants relative to wild type.

	Mutant background						
LPM pH 5.8	Δ*ssrB*	Δ*ompR*	Δ*slyA*	*phoP::Cm*	*fis::Cm*	Δ*hha*Δ*ydgT*	*hnsQ92am*
P*ssrA*	67±6.4	0.1±0.0	59±4.9	72±12	75±10	250±67	390±14
P*ssaB*	0.0±0.0	0.1±0.1	0.9±0.2	20±5.8	4.6±4.9	263±52	270±20
P*sseA*	0.2±0.0	0.8±0.1	1.2±0.3	37±9.4	12±8.3	296±37	247±25
P*ssaG*	15±1.5	12±2	11±1.2	36±4.8	10±4.3	26±10	173±14
P*ssaM*	2.9±0.2	3.1±0.5	3.4±0.2	24±5.8	9.9±3.0	1050±41	413±72
P*ssaR*	3.6±0.5	3.6±0.7	4.2±0.5	33±6.4	12±1.9	26±1.2	96±3.3
**M9-CAA**							
P*ssrA*	95±3.9	0.4±0.0	105±12	106±3.0	50±3.1	599±208	176±18
P*ssaG*	100±6.7	114±3.6	96±1.3	96±6.4	18±0.7	240±50	117±8.8

### SPI-2 promoters are induced in the lumen of the gut following oral infection

**Figure 2:**
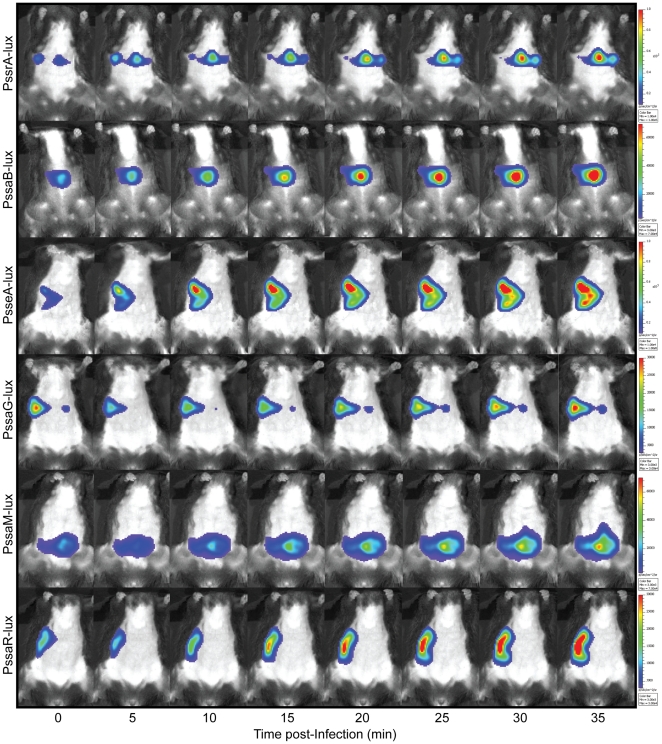
SPI-2 promoter activity increases immediately following entry into the small intestine. Mice were infected by oral gavage with *S*. Typhimurium strains carrying the luciferase transcriptional reporters. Animals were anesthetised and luminescence was measured as described in Methods. Colour bars for each reporter time course are indicated. Data is representative of four biological replicates each showing similar results.

**Figure 3:**
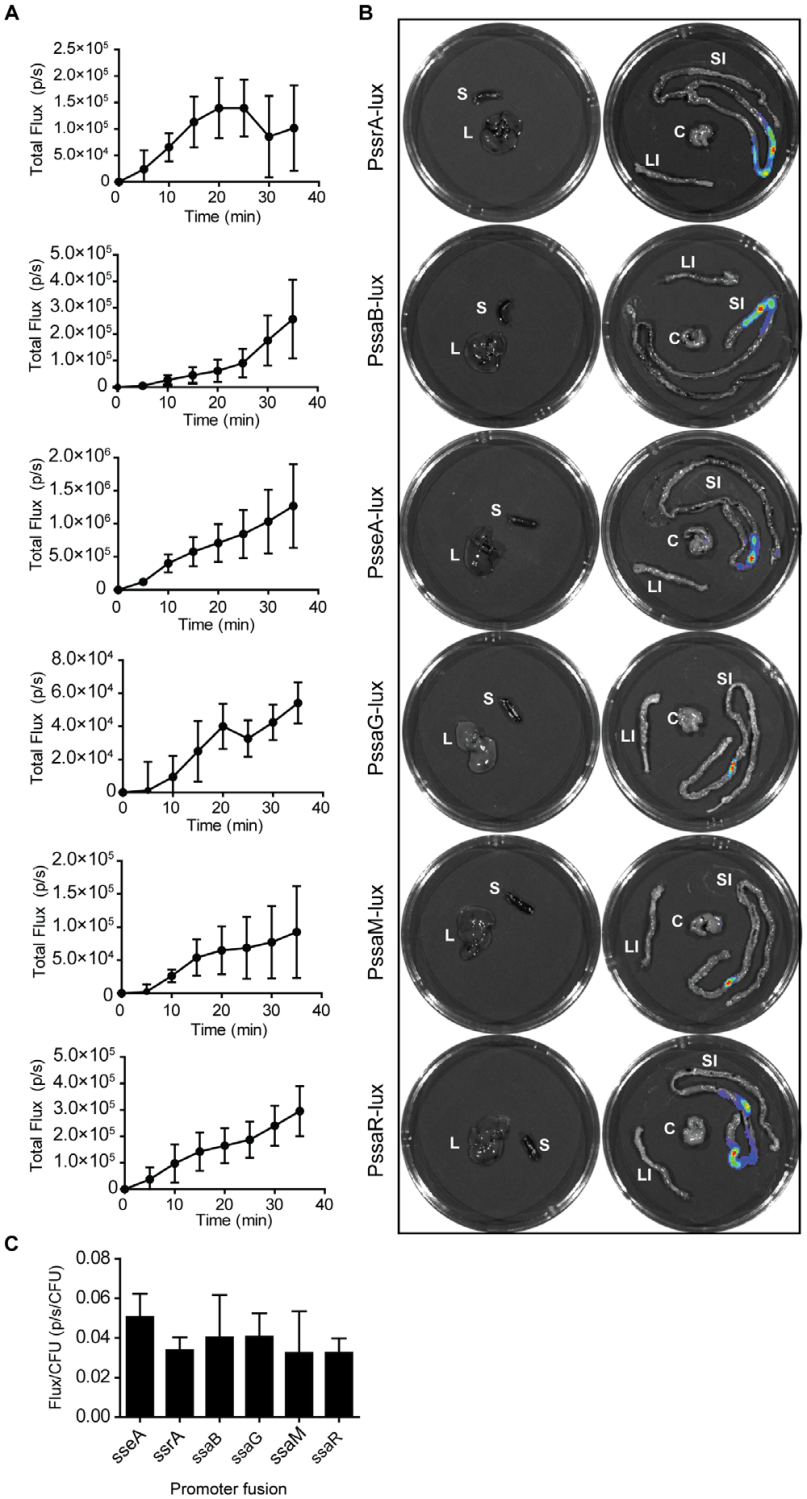
Quantification and ex vivo imaging of tissue luminescence. (**A**) Quantification of luminescence (total flux) is shown as the mean with standard deviation for each time point (*n* = 4). (**B**) Individual organs (S, spleen and L, liver, left panels; SI, small intestine; LI, large intestine and C, cecum, right panels) were imaged *ex vivo* at necropsy. (**C**) Light flux from individual organs was normalized to bacterial load. Data are the means with standard deviation from four organs for each reporter strain at the termination of the 35 min imaging session.

The observation that SPI-2 promoters are modestly active under non-inducing conditions suggested that extracellular priming of SPI-2 gene expression may occur. Previous work using recombinase-based *in vivo* expression had established that three promoters, (*sseA, ssaG*, and *ssaB/spiC*), were active in the lumen of the murine gut following direct injection of bacteria into ileal loops [27]. However, the *in vivo* activity of the entire complement of SPI-2 promoters following oral infection has not been tested. Mice infected by oral gavage with individual *Salmonella* strains that report the activity of each SPI-2 promoter were subjected to *in vivo* luminescence imaging immediately following infection (Figure 2). Each SPI-2 promoter was simultaneously and immediately activated with luciferase signal being localized exclusively to the small intestine in the first 35 min following infection, as determined by *ex vivo* imaging of individual organs at the terminal time point (Figure 3). When we compared the normalized light flux from each promoter, we found no significant difference in relative promoter activity, nor differences in the number of bacteria of each reporter strain recovered from each organ (data not shown). To assess promoter activity in animals over a longer time period, mice were imaged every day for three days following oral infection. These data showed that *sseA* promoter activity remained active over three days in bacteria localized in the gut (Figure S3). Using *ex vivo* imaging at necropsy we also detected luminescence signal originating from systemic tissues since *S.* Typhimurium gives rise to an invasive infection in mice (Figure S3).

**Figure 4:**
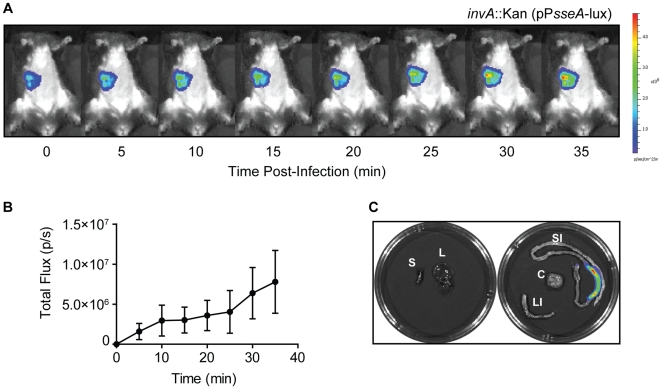
SPI-2 promoter activity in the small intestine does not require T3SS-1-mediated invasion. Mice were infected with an invasion-deficient mutant (*invA*::Kan) carrying an *sseA* promoter fusion to *luxCDABE*. Immediately after infection, anesthetised mice were imaged as described. (**A**) Whole-body luminescence from infected mice. Images are representative of three individual animals. (**B**) Quantification of luminescence (total flux) is shown as the mean with standard deviation for each time point (*n* = 3). (**C**) Individual organs (spleen and liver, left panel; small intestine, colon and cecum, right panel) were imaged *ex vivo* at necropsy.

The rapid increase in SPI-2 promoter activity observed following bacterial entry into the small intestine suggested that transcription was originating in the gut lumen prior to bacterial invasion. To investigate this, we constructed the P*sseA-lux* reporter in an *invA* mutant that is defective for cellular invasion [32] and quantified luminescence following oral infection. Promoter activity from the invasion-deficient strain showed a rapid increase after infection, similar in tempo and magnitude to that from wild type cells (Figure 4). As expected, luminescence was localized exclusively to the small intestine, suggesting that immune cell sampling of luminal bacteria was not responsible for this activity. These data are consistent with results using direct injection into murine ileal loops of recombinase-based reporter strains [27]. These results demonstrate that following entry of *S*. Typhimurium in to the intestinal lumen, all SPI-2 promoters undergo a rapid increase in activity that precedes cellular invasion.

### SPI-2 transcriptional priming does not require host cell contact

**Figure 5:**
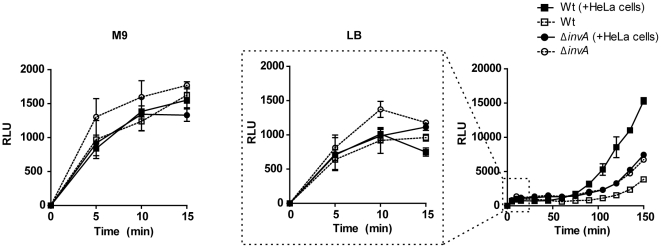
SPI-2 undergoes two stages of transcriptional up-regulation. Wild type or invasion deficient (Δ*invA*) *Salmonella* carrying *sseA* bioluminescence promoter fusions were pre-grown in M9-CAA or LB then sub-cultured into DMEM/10%FBS. Bioluminescence activity was monitored in the presence or absence of HeLa cells.

Our finding that SPI-2 promoters are rapidly induced following entry into the lumen but prior to invasion prompted us to question whether this activity was dependent on host cell contact. P*sseA*-*lux* reporter bacteria were pre-grown in either M9-CAA or LB then sub-cultured into DMEM/10%FBS and luminescence activity was recorded in 96-well plates in the presence or absence of HeLa cells. Plates were centrifuged to synchronize host cell contact. Regardless of the pre-growth media, P*sseA*-*lux* activity was found to have immediate transcriptional activity within the first 15 minutes that was independent of both invasion and the presence of HeLa cells (Figure 5). A second peak in transcriptional activity was observed at 1 hour post-infection which reflects activity in the intracellular niche. This data supports the model that SPI-2 undergoes two distinct transcriptional activation events; a pre-invasion priming activity and a transcriptional up-regulation specific to the intracellular niche.

## Discussion

Since the discovery of the T3SS-2 [6], [7], extensive work has elucidated its essential role for intracellular survival of *S*. Typhimurium. The regulation of this system has been well characterized for conditions that mimic the intracellular environment encountered by the bacteria following invasion. However little is known about the regulation of the SPI-2 T3SS preceding cellular invasion, although we think such a regulatory input would have relevance. *Salmonella s*urvival in macrophages and other cell types requires deployment of bacterial effectors by the SPI-2 T3SS that are known to block phagosome maturation and to counteract host defensive mechanisms such as reactive oxygen and nitrogen species [8], [9], [33], [34]. These processes are invoked immediately following phagocytosis, which would require a coincident functional response from the T3SS.

Although all SPI-2 promoters had simultaneous and high activity upon transfer to a synthetic inducing media, most – particularly the *ssaG* and *ssrA* promoters – had significant albeit lesser activity in non-inducing media. Surprisingly, the SPI-2 response regulator SsrB accounted for less than 5% of this activity and instead the transcriptional input was dominated by OmpR and Fis. Indeed, Fis binding sites have been identified upstream of *ssaG* [35] and OmpR and SsrB binding sites overlap at the *ssrA* promoter [14], which is entirely consistent with the transcriptional inputs we measured. Expression of SPI-2 immediately following entry of the bacteria into the small intestine is also consistent with a growing body of evidence indicating that the SPI-2 T3SS contributes to intestinal colonization. Using a recombinase-based reporter system and mouse ileal loops it was shown that the *sseA* promoter was activated within 15 min of entry into the ileum [27] when bacterial cells are associated with the apical surface of the host epithelium. This activity was also dependent on OmpR, suggesting that this regulatory input may be a key source of transcriptional priming *in vivo* prior to cellular invasion. Bovine and mouse infections have shown that the SPI-2 T3SS is necessary for enteric infection and triggers colitis in a MyD88-dependent manner [24], [25], [26], [36]. Our data in conjunction with these findings provides strong evidence for the expression of the SPI-2 T3SS in the intestine. It also implies that alternative extracellular signals are involved in SPI-2 regulation within the intestinal lumen, with possible candidates being mammalian body temperature [31], the acidity encountered during transit through the stomach, and other signals that are presently unknown.

The exact role of SPI-2 promoter activity in the intestinal lumen is presently unclear. Although SPI-2 is needed for enteric infection, this phenotype does not manifest until several days after infection, suggesting that the early transcriptional activity we measured is unrelated to this functionality. Instead, we propose that rapid activation of SPI-2 following entry into the lumen of the host gut reflects transcriptional priming needed for intracellular survival. Consistent with the notion of transcriptional priming, in mice in which disease is dominated by a systemic infection of the reticuloendothelial system, we found each promoter in SPI-2 to be active within five minutes following oral infection. This activity was sustained even in bacteria with a genetic lesion in the invasion machinery, indicating that SPI-2 transcriptional priming precedes cellular invasion. The T3SS-2 is needed for *Salmonella* to evade host antibacterial mechanisms such as reactive oxygen and nitrogen delivery to the nascent phagosome [8], [9] and SPI-2 mutant bacteria have a marked defect in preventing NADPH oxidase recruitment to the phagosome [37]. However, reactive oxygen generation inside nascent phagosomes by the host NADPH oxidase complex is detectable within 1-min following phagocytosis of reactive oxygen-sensitive beads [38] or yeast cells [39], which argues strongly for transcription priming of this bacterial defence system before the invasion event. Further research will be needed to quantify the intracellular fitness benefit immediately following invasion that is conferred by this early SPI-2 gene expression.

## Materials and Methods

### Ethics statement

All animal work was approved by the Animal Review Ethics Board at McMaster University under Animal Use Protocol #09-07-26, and conducted according to guidelines set by the Canadian Council on Animal Care.

### Bacterial strains and growth conditions

*Salmonella enterica* serovar Typhimurium strain SL1344 was used for all experiments and all mutants are derivatives thereof. Bacteria were grown at 37°C with aeration in the presence of selective antibiotics where appropriate as follows: ampicillin (100 µg/mL), kanamycin (50 µg/mL), chloramphenicol (34 µg/mL), tetracycline (12 µg/mL) and streptomycin (50 µg/mL). For transcriptional reporter experiments, bacteria were cultured overnight in M9-CAA minimal media (5 mM Na_2_HPO_4_·7H_2_O, 22 mM KH_2_PO_4_, 8.6 mM NaCl, 18.6 mM NH_4_Cl, 11.1 mM glucose, 2 mM MgSO_4_, 100 µM CaCl_2_, 0.1% casamino acids). Low phosphate, low magnesium medium (LPM) [21] pH 5.8 was used as a highly-inducing medium for SPI-2 gene expression (5 mM KCl, 7.5 mM (NH_4_)_2_SO_4_, 80 mM MES, 38 mM glycerol, 0.1% casamino acids, 24 µM MgCl_2_, 337 µM PO_4_^3−^).

### Cloning and mutant construction

Unmarked, in-frame deletions of *slyA* and *ompR* as well as a marked in-frame deletion of *fis* (*fis*::Kan) were constructed using Lambda red recombination [40]. Transcriptional reporters with *luxCDABE* were constructed in pGEN-*luxCDABE* [41] for all six of the promoters identified in SPI-2 [11] including *ssrA*, *ssaB*, *sseA*, *ssaG*, *ssaM*, and *ssaR*. All primers used for mutant construction and cloning of transcriptional reporters are listed in Table S2.

### Transcriptional reporter assays

Bacteria were grown overnight in M9 CAA at 37°C with shaking and then sub-cultured 1∶100 into M9 CAA in 96-well plates (Costar). Bacteria were grown at 37°C (150 rpm) and optical density at 600 nm (OD_600_) and luminescence were measured every 15 min using an Envison 2104 plate reader (PerkinElmer). Luminescence data was normalized to OD_600 nm_ for each time point and adjusted to the luminescence at time zero.

### *In vivo* bioluminescence imaging

Three days prior to infection, abdominal fur was removed from the mice using a depilatory cream. *Salmonella* with luciferase reporters were grown overnight in M9 CAA with selective antibiotics at 37°C. Bacteria were washed twice and resuspended in 0.1 M HEPES (pH 8.0), 0.9% NaCl. Female C57BL/6 mice (Jackson Laboratories) were infected by oral gavage with ∼10^8^ live bacteria. Animals were immediately anaesthetized with 2% isofluorane carried in 2% oxygen and imaged dorsally in an IVIS Spectrum (Caliper Life Sciences). Greyscale and luminescence images were captured at 5 min intervals for 35 min and processed using Living Image Software. After the imaging session, mice were sacrificed and individual organs were imaged *ex vivo* and then processed for bacterial load determination by homogenization in a Mixer Mill (Retsch; Haan, Germany) and selective plating on solid media. Total flux was normalized to the initial flux recorded at time zero.

### HeLa cell culture

HeLa cells were seeded in black 96-well plates with clear bottoms at 2×10^5^ cells/mL 24 h prior to infection. Overnight cultures of wild type or an invasion-deficient Δ*invA* strain, both carrying (pPsseA-lux) were pre-grown in LB or M9-CAA for 3 hours then sub-cultured 1∶100 into DMEM (Gibco) with 10% fetal bovine serum (FBS). 50 µL was added to each well and centrifuged at 500 x *g* for 5 min. Bioluminescence was recorded as described above. Cells were grown at 37°C in 5% CO_2_.

## Supporting Information

Figure S1 **SPI-2 expression in inducing versus non-inducing conditions has distinct regulatory inputs.** Graphs represent the entire dataset collected for the experiments involving transcriptional activators summarized in Table 1. Wild type *S*. Typhimurium carrying luciferase transcriptional reporters for each SPI-2 promoter were sub-cultured from actively growing cultures in M9-CAA into either inducing (LPM pH 5.8) or non-inducing (M9-CAA) media. Luminescence was measured continuously and normalized to OD_600 nm_ at each time point (*n* = 12). Data are the means with standard deviation.
(TIF)

Figure S2 **SPI-2 expression in inducing versus non-inducing conditions for transcriptional repressor mutants.** Graphs represent the entire dataset collected for the experiments involving transcriptional repressors summarized in Table 1. Wild type *S*. Typhimurium carrying luciferase transcriptional reporters for each SPI-2 promoter were sub-cultured from actively growing cultures in M9-CAA into either inducing (LPM pH 5.8) or non-inducing (M9-CAA) media. Luminescence was measured continuously and normalized to OD_600 nm_ at each time point (*n* = 12). Data are the means with standard deviation.
(TIF)

Figure S3 **The** ***sseA*** **promoter remains active from 1 to 3 days post infection.** Mice were infected with wild type *Salmonella* containing the *sseA* transcriptional reporter. **(A)** Luminescence images were acquired every 24 h and are representative of three individuals animals. **(B)** Total flux from whole-animal imaging was quantified and is shown as the mean with standard deviation (*n* = 3). **(C)** At 3 days post-infection organs from infected mice from (A) were imaged *ex vivo* (S, spleen; L, liver; C, cecum; SI, small intestine; LI, large intestine).
(TIF)

Table S1 **Transcriptional reporter data for all SPI-2 promoters in wild type** ***Salmonella*** **and seven regulator mutants.** Experiments were conducted as described in Materials and Methods and data is shown as the mean with standard deviation from three separate experiments.
(PDF)

Table S2 **List of primers and their sequences used for construction of mutants and transcriptional reporters.**
(DOC)
