# Supplementary figures and images for: Transcriptional Priming of Salmonella Pathogenicity Island-2 Precedes Cellular Invasion
Source: PLoS One. 2011 Jun 28;6(6):e21648. doi: 10.1371/journal.pone.0021648 (PMC3125303; doi:10.1371/journal.pone.0021648)

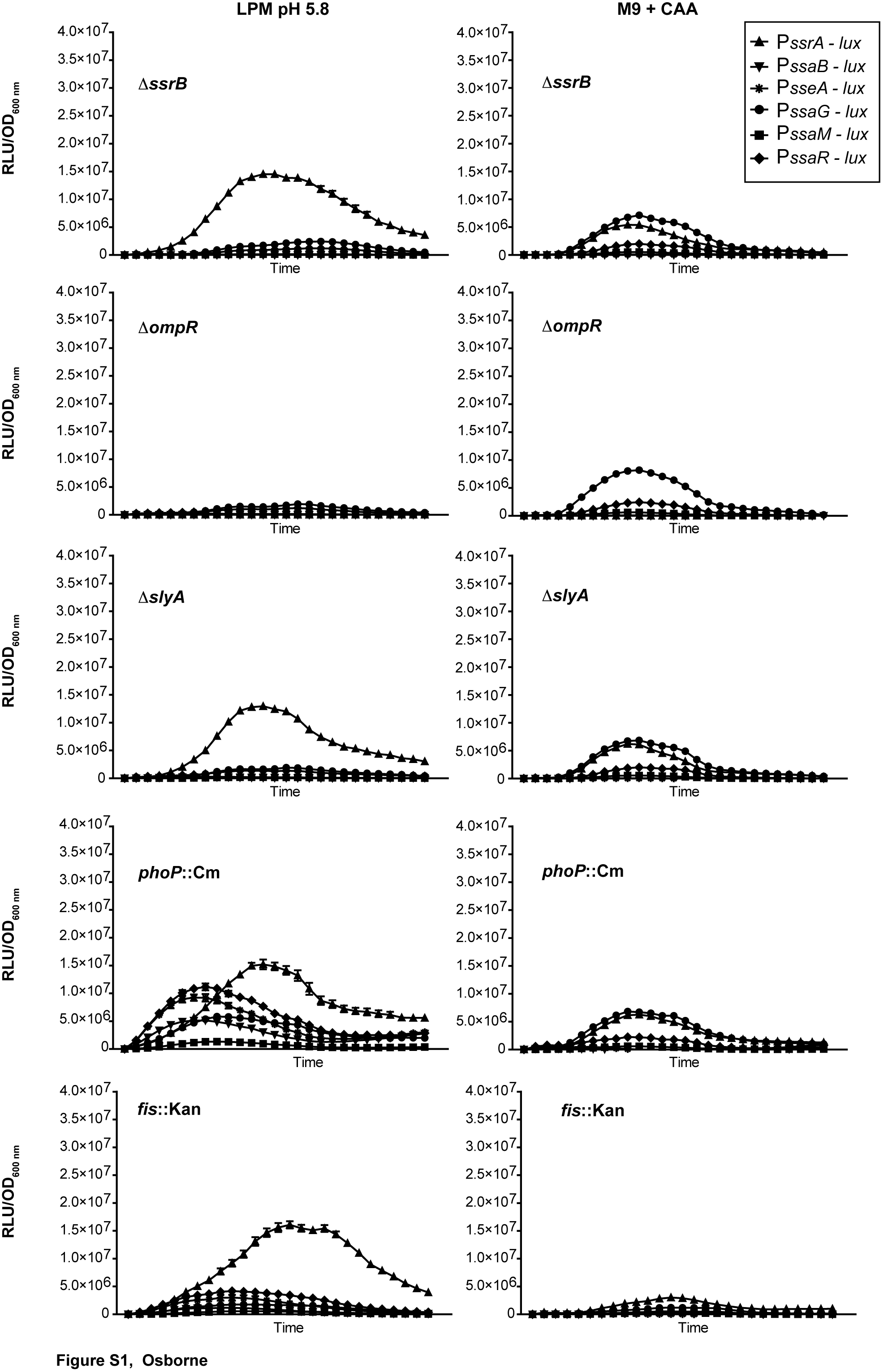

Supplement: Figure S1 — SPI-2 expression in inducing versus non-inducing conditions has distinct regulatory inputs. Graphs represent the entire dataset collected for the experiments involving transcriptional activators summarized in Table 1. Wild type S. Typhimurium carrying luciferase transcriptional reporters for each SPI-2 promoter were sub-cultured from actively growing cultures in M9-CAA into either inducing (LPM pH 5.8) or non-inducing (M9-CAA) media. Luminescence was measured continuously and normalized to OD600 nm at each time point (n = 12). Data are the means with standard deviation. (TIF) [file pone.0021648.s001.tif]

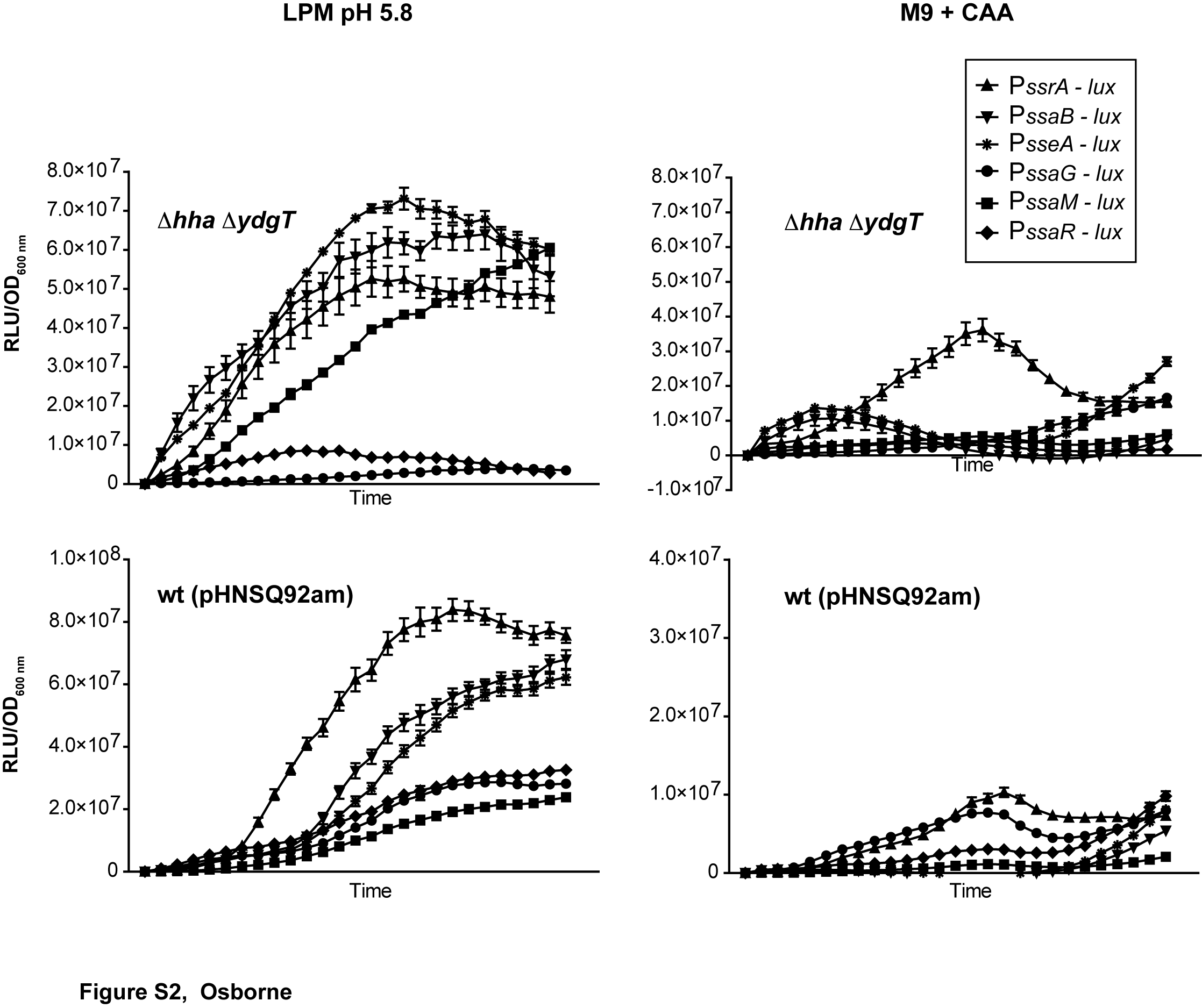

Supplement: Figure S2 — SPI-2 expression in inducing versus non-inducing conditions for transcriptional repressor mutants. Graphs represent the entire dataset collected for the experiments involving transcriptional repressors summarized in Table 1. Wild type S. Typhimurium carrying luciferase transcriptional reporters for each SPI-2 promoter were sub-cultured from actively growing cultures in M9-CAA into either inducing (LPM pH 5.8) or non-inducing (M9-CAA) media. Luminescence was measured continuously and normalized to OD600 nm at each time point (n = 12). Data are the means with standard deviation. (TIF) [file pone.0021648.s002.tif]

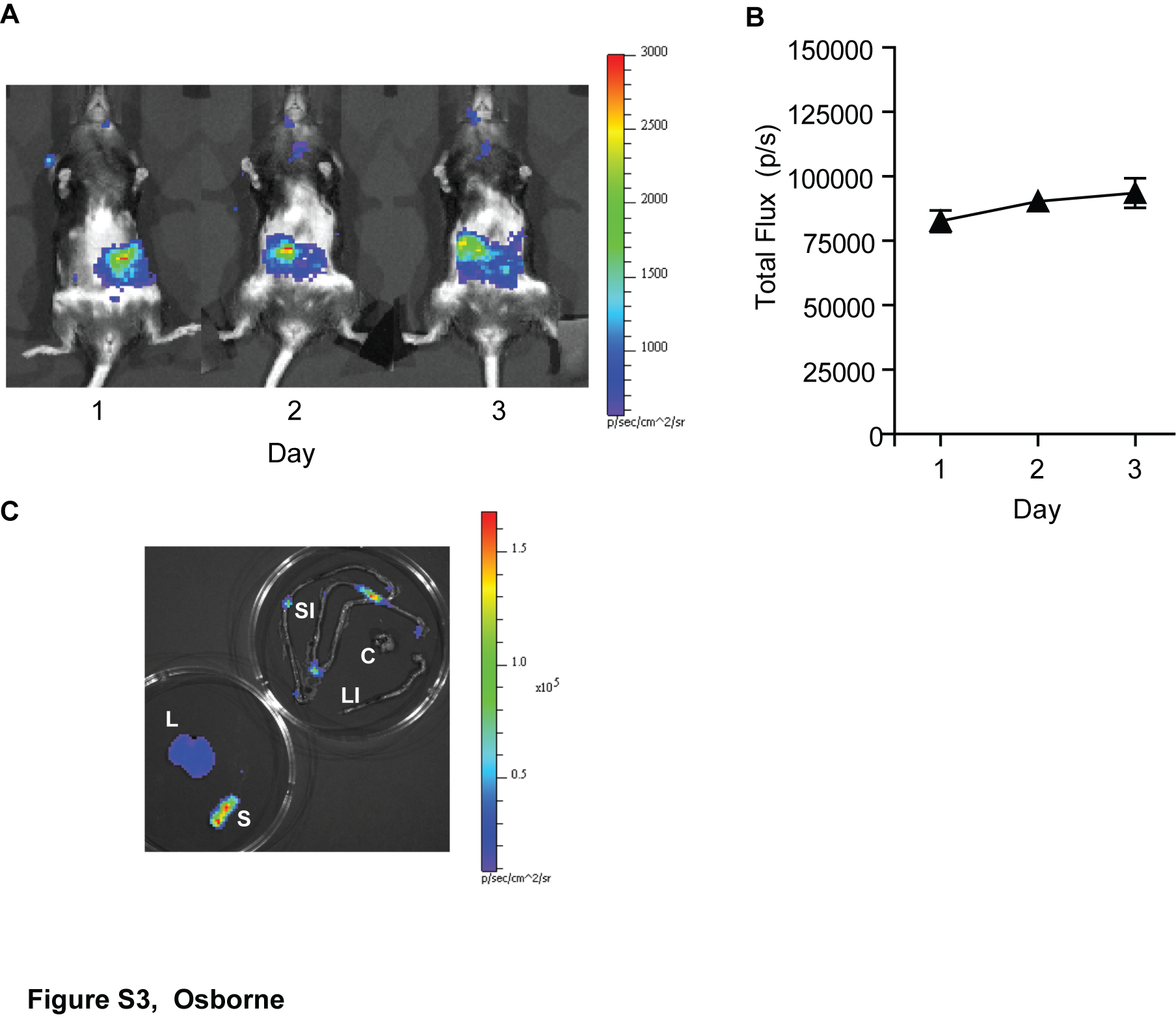

Supplement: Figure S3 — The sseA promoter remains active from 1 to 3 days post infection. Mice were infected with wild type Salmonella containing the sseA transcriptional reporter. (A) Luminescence images were acquired every 24 h and are representative of three individuals animals. (B) Total flux from whole-animal imaging was quantified and is shown as the mean with standard deviation (n = 3). (C) At 3 days post-infection organs from infected mice from (A) were imaged ex vivo (S, spleen; L, liver; C, cecum; SI, small intestine; LI, large intestine). (TIF) [file pone.0021648.s003.tif]
