# Supplementary material for: Transcriptional Priming of Salmonella Pathogenicity Island-2 Precedes Cellular Invasion
Source: PLoS One. 2011 Jun 28;6(6):e21648. doi: 10.1371/journal.pone.0021648 (PMC3125303; doi:10.1371/journal.pone.0021648)
Supplement: Table S1 — Transcriptional reporter data for all SPI-2 promoters in wild type Salmonella and seven regulator mutants. Experiments were conducted as described in Materials and Methods and data is shown as the mean with standard deviation from three separate experiments. (PDF) [file pone.0021648.s004.pdf]

Wild type

Promoter LPM pH 5.8	Rate of Luminescence Increase (RLU/min)	Max. Instantaneous Rate of Luminescence Increase (RLU/min)	Time of Max. Instantaneous Increase Rate (min)	Max. Luminescence (RLU/OD600nm)	Time of Max. Luminescence (min)	Steady State % of Max.
ssrA	$1.9 \times 10^5 \pm 1.6 \times 10^4$	$2.5 \times 10^5 \pm 2.3 \times 10^4$	115 ± 9	$2.2 \times 10^7 \pm 2.4 \times 10^6$	180 ± 15	24 ± 4
ssaB	$2.0 \times 10^5 \pm 2.0 \times 10^4$	$2.4 \times 10^5 \pm 2.3 \times 10^4$	115 ± 22	$2.5 \times 10^7 \pm 3.1 \times 10^6$	165 ± 30	29 ± 2
sseA	$1.8 \times 10^5 \pm 3.8 \times 10^4$	$2.0 \times 10^5 \pm 3.6 \times 10^4$	85 ± 38	$2.5 \times 10^7 \pm 1.5 \times 10^6$	170 ± 38	33 ± 4
ssaG	$1.4 \times 10^5 \pm 2.4 \times 10^4$	$1.8 \times 10^5 \pm 3. \times 10^4$	95 ± 23	$1.7 \times 10^7 \pm 4.3 \times 10^5$	185 ± 46	25 ± 5
ssaM	$5.7 \times 10^4 \pm 1.1 \times 10^4$	$6.7 \times 10^4 \pm 1.2 \times 10^4$	110 ± 17	$5.8 \times 10^6 \pm 6.1 \times 10^5$	190 ± 48	33 ± 1
ssaR	$2.3 \times 10^5 \pm 6.4 \times 10^4$	$3.2 \times 10^5 \pm 1.1 \times 10^5$	100 ± 17	$3.4 \times 10^7 \pm 3.3 \times 10^6$	180 ± 40	25 ± 3
Promoter M9						
ssrA	$8.8 \times 10^4 \pm 6.2 \times 10^3$	$1.0 \times 10^5 \pm 1.2 \times 10^4$	70 ± 9 **	$6.1 \times 10^6 \pm 3.5 \times 10^5$	130 ± 9	N/A
ssaG	$8.1 \times 10^4 \pm 3.5 \times 10^3$	$1.0 \times 10^5 \pm 1.2 \times 10^4$	70 ± 9	$7.2 \times 10^6 \pm 4.7 \times 10^5$	135 ± 0	N/A

ΔssrB

Promoter LPM pH 5.8	Rate of Luminescence Increase (RLU/min)	Max. Instantaneous Rate of Luminescence Increase (RLU/min)	Time of Max. Instantaneous Increase Rate (min)	Max. Luminescence (RLU/OD600nm)	Time of Max. Luminescence (min)	Steady State % of Max.
ssrA	$1.6 \times 10^5 \pm 2.3 \times 10^4$	$1.8 \times 10^5 \pm 1.6 \times 10^4$	115 ± 17	$1.5 \times 10^7 \pm 1.4 \times 10^6$	185 ± 9	26 ± 1
ssaB	$2.6 \times 10^1 \pm 1.3 \times 10^1$	$1.9 \times 10^2 \pm 4.4 \times 10^1$	140 ± 17	$5.1 \times 10^3 \pm 1.9 \times 10^3$	200 ± 17	0
sseA	$3.0 \times 10^2 \pm 1.1 \times 10^2$	$9.1 \times 10^2 \pm 2.7 \times 10^2$	90 ± 65	$5.4 \times 10^4 \pm 6.2 \times 10^3$	225 ± 15	11 ± 3
ssaG	$1.4 \times 10^4 \pm 1.6 \times 10^3$	$2.8 \times 10^2 \pm 5.3 \times 10^3$	160 ± 46	$2.5 \times 10^6 \pm 2.5 \times 10^5$	245 ± 17	22 ± 6
ssaM	$2.0 \times 10^3 \pm 4.3 \times 10^2$	$2.5 \times 10^3 \pm 7.1 \times 10^2$	135 ± 15	$1.7 \times 10^5 \pm 8.7 \times 10^3$	215 ± 23	17 ± 9
ssaR	$9.7 \times 10^3 \pm 4.8 \times 10^3$	$1.7 \times 10^4 \pm 3.6 \times 10^3$	130 ± 9	$1.2 \times 10^6 \pm 1.6 \times 10^5$	250 ± 17	19 ± 8
Promoter M9						
ssrA	$7.8 \times 10^4 \pm 9.1 \times 10^3$	$9.3 \times 10^4 \pm 1.4 \times 10^4$	105 ± 0	$5.8 \times 10^6 \pm 2.4 \times 10^5$	140 ± 17	N/A
ssaG	$8.0 \times 10^4 \pm 1.6 \times 10^4$	$9.8 \times 10^4 \pm 2.0 \times 10^4$	95 ± 17	$7.2 \times 10^6 \pm 4.8 \times 10^5$	145 ± 9	N/A

ΔompR

Promoter LPM pH 5.8	Rate of Luminescence Increase (RLU/min)	Max. Instantaneous Rate of Luminescence Increase (RLU/min)	Time of Max. Instantaneous Increase Rate (min)	Max. Luminescence (RLU/OD600nm)	Time of Max. Luminescence (min)	Steady State % of Max.
ssrA	$6.0 \times 10^1 \pm 1.1 \times 10^1$	$3.8 \times 10^2 \pm 1.1 \times 10^2$	120 ± 15	$1.4 \times 10^4 \pm 2.3 \times 10^3$	195 ± 54	0.3 ± 0.1
ssaB	$5.0 \times 10^2 \pm 1.4 \times 10^1$	$8.3 \times 10^2 \pm 1.5 \times 10^2$	45 ± 15	$3.2 \times 10^4 \pm 2.0 \times 10^4$	145 ± 83	0.0 ± 0.0
sseA	$2.5 \times 10^3 \pm 4.5 \times 10^2$	$3.5 \times 10^3 \pm 4.3 \times 10^2$	50 ± 9	$2.1 \times 10^5 \pm 3.2 \times 10^4$	105 ± 0	0.0 ± 0.0
ssaG	$1.2 \times 10^4 \pm 1.7 \times 10^3$	$2.6 \times 10^4 \pm 1.2 \times 10^3$	125 ± 9	$2.0 \times 10^6 \pm 3.0 \times 10^5$	230 ± 9	0.2 ± 0.0
ssaM	$2.0 \times 10^3 \pm 2.8 \times 10^2$	$3.1 \times 10^3 \pm 3.1 \times 10^2$	130 ± 9	$1.8 \times 10^5 \pm 2.7 \times 10^4$	145 ± 9	0.2 ± 0.0
ssaR	$6.5 \times 10^3 \pm 1.2 \times 10^3$	$1.4 \times 10^4 \pm 1.3 \times 10^3$	90 ± 65	$1.2 \times 10^6 \pm 2.4 \times 10^5$	225 ± 15	0.2 ± 0.0
Promoter M9						
ssrA	$2.8 \times 10^2 \pm 5.9 \times 10^1$	$4.2 \times 10^2 \pm 5.3 \times 10^1$	90 ± 26	$2.3 \times 10^4 \pm 1.2 \times 10^3$	120 ± 26	N/A
ssaG	$9.7 \times 10^4 \pm 8.7 \times 10^3$	$1.2 \times 10^5 \pm 1.2 \times 10^4$	80 ± 9	$8.2 \times 10^6 \pm 2.6 \times 10^5$	150 ± 0	N/A

ΔslyA

Promoter LPM pH 5.8	Rate of Luminescence Increase (RLU/min)	Max. Instantaneous Rate of Luminescence Increase (RLU/min)	Time of Max. Instantaneous Increase Rate (min)	Max. Luminescence (RLU/OD600nm)	Time of Max. Luminescence (min)	Steady State % of Max.
ssrA	$1.4 \times 10^5 \pm 1.1 \times 10^4$	$1.8 \times 10^5 \pm 4.1 \times 10^3$	125 ± 9	$1.3 \times 10^7 \pm 1.1 \times 10^6$	180 ± 0	26 ± 4
ssaB	$3.4 \times 10^3 \pm 8.1 \times 10^2$	$4.8 \times 10^3 \pm 1.2 \times 10^3$	50 ± 9	$2.3 \times 10^5 \pm 6.0 \times 10^4$	105 ± 15	26 ± 2
sseA	$4.2 \times 10^3 \pm 1.2 \times 10^3$	$5.6 \times 10^3 \pm 1.4 \times 10^3$	40 ± 9	$3.1 \times 10^5 \pm 7.7 \times 10^4$	85 ± 9	20 ± 3
ssaG	$1.8 \times 10^4 \pm 5.1 \times 10^3$	$2.7 \times 10^4 \pm 6.7 \times 10^3$	120 ± 0	$1.9 \times 10^6 \pm 2.0 \times 10^5$	215 ± 9	26 ± 13
ssaM	$2.4 \times 10^3 \pm 3.5 \times 10^2$	$3.2 \times 10^3 \pm 9.8 \times 10^2$	120 ± 0	$2.0 \times 10^5 \pm 1.2 \times 10^4$	160 ± 9	19 ± 4
ssaR	$1.0 \times 10^4 \pm 2.9 \times 10^3$	$1.9 \times 10^4 \pm 2.4 \times 10^3$	85 ± 61	$1.4 \times 10^6 \pm 1.7 \times 10^5$	195 ± 40	30 ± 8
Promoter M9						
ssrA	$8.1 \times 10^4 \pm 4.6 \times 10^3$	$1.1 \times 10^5 \pm 8.0 \times 10^3$	90 ± 0	$6.4 \times 10^6 \pm 7.2 \times 10^5$	140 ± 9	N/A
ssaG	$8.8 \times 10^4 \pm 1.1 \times 10^4$	$1.1 \times 10^5 \pm 1.8 \times 10^4$	90 ± 0	$6.9 \times 10^6 \pm 9.1 \times 10^4$	145 ± 9	N/A

***phoP*::Cm**

Promoter LPM pH 5.8	Rate of Luminescence Increase (RLU/min)	Max. Instantaneous Rate of Luminescence Increase (RLU/min)	Instantaneous Increase Rate (min)	Max. Luminescence (RLU/OD600nm)	Time of Max. Luminescence (min)	Steady State % of Max.
ssrA	$1.3 \times 10^{5} \pm 2.0 \times 10^{4}$	$1.7 \times 10^{5} \pm 2.4 \times 10^{4}$	125 ± 23	$1.6 \times 10^{7} \pm 2.3 \times 10^{6}$	175 ± 9	35 ± 5
ssaB	$7.3 \times 10^{4} \pm 2.3 \times 10^{4}$	$8.7 \times 10^{4} \pm 2.5 \times 10^{4}$	35 ± 9	$5.2 \times 10^{6} \pm 1.5 \times 10^{6}$	95 ± 9	58 ± 33
sseA	$1.3 \times 10^{5} \pm 3.8 \times 10^{4}$	$1.7 \times 10^{5} \pm 4.0 \times 10^{4}$	30 ± 0	$9.4 \times 10^{6} \pm 2.4 \times 10^{6}$	95 ± 9	34 ± 21
ssaG	$5.5 \times 10^{4} \pm 9.2 \times 10^{3}$	$7.2 \times 10^{4} \pm 6.6 \times 10^{3}$	70 ± 9	$6.0 \times 10^{6} \pm 8.1 \times 10^{5}$	130 ± 17	36 ± 19
ssaM	$1.6 \times 10^{4} \pm 3.8 \times 10^{3}$	$2.0 \times 10^{4} \pm 6.0 \times 10^{3}$	75 ± 15	$1.4 \times 10^{6} \pm 3.4 \times 10^{5}$	115 ± 9	28 ± 17
ssaR	$1.4 \times 10^{5} \pm 2.3 \times 10^{4}$	$1.7 \times 10^{5} \pm 2.7 \times 10^{4}$	30 ± 0	$1.1 \times 10^{7} \pm 2.2 \times 10^{6}$	100 ± 9	26 ± 15
Promoter M9						
ssrA	$7.6 \times 10^{4} \pm 5.5 \times 10^{3}$	$8.9 \times 10^{4} \pm 9.8 \times 10^{3}$	95 ± 9	$6.4 \times 10^{6} \pm 1.8 \times 10^{5}$	145 ± 9	N/A
ssaG	$8.0 \times 10^{4} \pm 3.0 \times 10^{3}$	$1.0 \times 10^{5} \pm 3.1 \times 10^{3}$	85 ± 9	$6.9 \times 10^{6} \pm 4.6 \times 10^{5}$	140 ± 9	N/A

***fis*::Kan**

Promoter LPM pH 5.8	Rate of Luminescence Increase (RLU/min)	Max. Instantaneous Rate of Luminescence Increase (RLU/min)	Time of Max. Instantaneous Increase Rate (min)	Max. Luminescence (RLU/OD600nm)	Time of Max. Luminescence (min)	Steady State % of Max.
ssrA	$1.0 \times 10^{5} \pm 1.6 \times 10^{4}$	$1.5 \times 10^{5} \pm 2.6 \times 10^{4}$	165 ± 0	$1.6 \times 10^{7} \pm 2.3 \times 10^{6}$	215 ± 23	26 ± 2
ssaB	$1.6 \times 10^{4} \pm 1.6 \times 10^{4}$	$1.7 \times 10^{4} \pm 1.8 \times 10^{4}$	55 ± 9	$1.2 \times 10^{6} \pm 1.3 \times 10^{6}$	105 ± 15	16 ± 8
sseA	$3.5 \times 10^{4} \pm 2.7 \times 10^{4}$	$4.3 \times 10^{4} \pm 3.0 \times 10^{4}$	50 ± 9	$3.0 \times 10^{6} \pm 2.1 \times 10^{6}$	135 ± 0	9 ± 2
ssaG	$1.8 \times 10^{4} \pm 1.1 \times 10^{4}$	$2.5 \times 10^{4} \pm 1.1 \times 10^{4}$	65 ± 9	$1.8 \times 10^{6} \pm 7.3 \times 10^{5}$	150 ± 15	18 ± 1
ssaM	$5.2 \times 10^{3} \pm 1.7 \times 10^{3}$	$7.1 \times 10^{3} \pm 2.7 \times 10^{3}$	85 ± 17	$5.7 \times 10^{5} \pm 1.7 \times 10^{5}$	155 ± 17	10 ± 1
ssaR	$4.5 \times 10^{4} \pm 1.1 \times 10^{4}$	$5.7 \times 10^{4} \pm 1.6 \times 10^{4}$	65 ± 9	$4.2 \times 10^{6} \pm 6.4 \times 10^{5}$	140 ± 9	10 ± 0
Promoter M9						
ssrA	$2.3 \times 10^{4} \pm 1.1 \times 10^{3}$	$3.3 \times 10^{4} \pm 4.0 \times 10^{3}$	105 ± 15	$3.0 \times 10^{6} \pm 1.9 \times 10^{5}$	180 ± 0	N/A
ssaG	$8.5 \times 10^{3} \pm 6.7 \times 10^{2}$	$1.3 \times 10^{4} \pm 2.3 \times 10^{2}$	135 ± 52	$1.3 \times 10^{6} \pm 5.3 \times 10^{4}$	195 ± 26	N/A

Δ*hha* Δ*ydgT*

Promoter LPM pH 5.8	Rate of Luminescence Increase (RLU/min)	Max. Instantaneous Rate of Luminescence Increase (RLU/min)	Time of Max. Instantaneous Increase Rate (min)	Max. Luminescence (RLU/OD600nm)	Time of Max. Luminescence (min)	Steady State % of Max.
ssrA	$3.9 \times 10^{5} \pm 1.9 \times 10^{5}$	$4.9 \times 10^{5} \pm 1.9 \times 10^{5}$	140 ± 87	$5.5 \times 10^{7} \pm 1.5 \times 10^{7}$	225 ± 26	88 ± 6
ssaB	$4.5 \times 10^{5} \pm 2.3 \times 10^{5}$	$6.2 \times 10^{5} \pm 2.2 \times 10^{5}$	150 ± 108	$6.7 \times 10^{7} \pm 1.3 \times 10^{7}$	270 ± 45	80 ± 15
sseA	$3.7 \times 10^{5} \pm 2.5 \times 10^{4}$	$5.3 \times 10^{5} \pm 5.8 \times 10^{4}$	175 ± 61	$7.5 \times 10^{7} \pm 9.2 \times 10^{6}$	225 ± 15	82 ± 4
ssaG	$1.6 \times 10^{4} \pm 6.2 \times 10^{3}$	$3.4 \times 10^{4} \pm 1.8 \times 10^{4}$	270 ± 0	$4.3 \times 10^{6} \pm 1.7 \times 10^{6}$	340 ± 61	N/A
ssaM	$1.9 \times 10^{5} \pm 2.3 \times 10^{4}$	$3.6 \times 10^{5} \pm 2.7 \times 10^{4}$	170 ± 69	$6.1 \times 10^{7} \pm 2.3 \times 10^{6}$	375 ± 0	N/A
ssaR	$5.5 \times 10^{4} \pm 1.7 \times 10^{3}$	$1.2 \times 10^{5} \pm 2.4 \times 10^{4}$	15 ± 0	$8.9 \times 10^{6} \pm 4.1 \times 10^{5}$	200 ± 62	35 ± 20
Promoter M9						
ssrA	$2.1 \times 10^{5} \pm 7.8 \times 10^{4}$	$3.0 \times 10^{5} \pm 8.3 \times 10^{4}$	125 ± 17	$3.6 \times 10^{7} \pm 1.3 \times 10^{7}$	220 ± 17	N/A
ssaG	$1.2 \times 10^{5} \pm 2.5 \times 10^{4}$	$1.7 \times 10^{5} \pm 2.7 \times 10^{3}$	340 ± 61	$1.7 \times 10^{7} \pm 3.5 \times 10^{6}$	360 ± 26	N/A

Wild type (pHNSQ92am)

Promoter LPM pH 5.8	Rate of Luminescence Increase (RLU/min)	Max. Instantaneous Rate of Luminescence Increase (RLU/min)	Time of Max. Instantaneous Increase Rate (min)	Max. Luminescence (RLU/OD600nm)	Time of Max. Luminescence (min)	Steady State % of Max.
ssrA	$4.9 \times 10^{5} \pm 6.4 \times 10^{4}$	$7.1 \times 10^{5} \pm 1.2 \times 10^{5}$	230 ± 38	$8.5 \times 10^{7} \pm 3.0 \times 10^{6}$	285 ± 30	N/A
ssaB	$3.5 \times 10^{5} \pm 1.7 \times 10^{5}$	$6.2 \times 10^{5} \pm 9.0 \times 10^{5}$	200 ± 23	$6.8 \times 10^{7} \pm 5.0 \times 10^{6}$	385 ± 9	N/A
sseA	$3.0 \times 10^{5} \pm 8.6 \times 10^{4}$	$5.3 \times 10^{5} \pm 9.9 \times 10^{4}$	250 ± 23	$6.3 \times 10^{7} \pm 6.2 \times 10^{6}$	385 ± 9	N/A
ssaG	$1.6 \times 10^{5} \pm 3.6 \times 10^{4}$	$2.9 \times 10^{5} \pm 6.1 \times 10^{4}$	230 ± 38	$2.9 \times 10^{7} \pm 2.4 \times 10^{6}$	330 ± 15	N/A
ssaM	$1.2 \times 10^{5} \pm 1.2 \times 10^{4}$	$1.8 \times 10^{5} \pm 1.4 \times 10^{4}$	230 ± 38	$2.4 \times 10^{7} \pm 4.2 \times 10^{6}$	390 ± 0	N/A
ssaR	$1.5 \times 10^{5} \pm 1.1 \times 10^{4}$	$2.6 \times 10^{5} \pm 6.2 \times 10^{4}$	230 ± 38	$3.3 \times 10^{7} \pm 1.1 \times 10^{6}$	385 ± 9	N/A
Promoter M9						
ssrA	$7.4 \times 10^{4} \pm 1.3 \times 10^{4}$	$1.3 \times 10^{5} \pm 3.1 \times 10^{4}$	210 ± 15	$1.1 \times 10^{7} \pm 1.1 \times 10^{6}$	245 ± 9	N/A
ssaG	$4.6 \times 10^{4} \pm 3.8 \times 10^{3}$	$7.5 \times 10^{4} \pm 2.1 \times 10^{4}$	265 ± 95	$8.4 \times 10^{6} \pm 6.3 \times 10^{5}$	275 ± 100	N/A
