# Supplementary material for: Transcriptional Priming of Salmonella Pathogenicity Island-2 Precedes Cellular Invasion
Source: PLoS One. 2011 Jun 28;6(6):e21648. doi: 10.1371/journal.pone.0021648 (PMC3125303; doi:10.1371/journal.pone.0021648)
Supplement: Table S2 — List of primers and their sequences used for construction of mutants and transcriptional reporters. (DOC) [file pone.0021648.s005.doc]

**Table S2. List of primers**

| **Construct** | **Name** | **Sequence** |
| --- | --- | --- |
| Δ*slyA* | SEO001 | gcaagctaattataaggagatgaaattggaatcgccactagtgtag  gctggagctgcttcg |
|  | SEO002 | ggccacacgtatgcccctgcacctcaatcgtgagagtgcaacatat  gaatatcctcctta |
| Δ*ompR* | SEO013 | gttgcgaacctttgggagtacagacaatgcaagagaattataaggtg  taggctggagctgcttcg |
|  | SEO014 | cttcgcggtgagaagcgcattcgcctcatgctttagaaccgtccatatg  aatatcctcctta |
| *fis*::Kan | SEO147 | agaaataaagagctgacagaactatgttcgaacaacgcgtagtgtag  gctggagctgcttcg |
|  | SEO148 | aacaagcagttagctaatcgaaattagttcatgccgtatttcatatgaata  tcctcctta |
| pP*ssrA*-*luxCDABE* | SEO135 | cgatacgtattacagccaaataattattgttgt |
|  | SEO136 | cgggatcctctggcataaagggtgaagt |
| pP*ssaB*-*luxCDABE* | SEO123 | cgggatccgtgccatcctttgccgttt |
|  | SEO124 | cgatacgtattacatgaatccctcctcagacat |
| pP*sseA*-*luxCDABE* | SEO129 | ggggtaccgcaaggttcaaccattacttg |
|  | SEO130 | cgatacgtattacgcagcctttttctttatca |
| pP*ssaG*-*luxCDABE* | SEO121 | cgggatccatatggagagtggtagaatag |
|  | SEO122 | cgatacgtattataattgtgcaatatccataa |
| pP*ssaM*-*luxCDABE* | SEO119 | cgggatccaatcaggttttattctgatacctgg |
|  | SEO120 | cgatacgtattaaatgagatcccaatccatcct |
| pP*ssaR*-*luxCDABE* | SEO137 | cgggatccccacaacaggtgctctttga |
|  | SEO138 | cgatacgtattatcgataaggcgctaatgctt |
